# 

**Published:** 1900-08-25

**Authors:** 


					THE HOSPITAL. "The standard of highest parity."?Lancet, August 25th, 1900.
CADBURY'S U ^ CADBURY'S
COCOA mm COCOA
ABSOLUTELY POU. KO DRUGS OR ALKALIES /U8$Q>nA/j
No. *26. Vol. xxvm. [ffS?J?] Saturday,
August 25, 1900.
[Sub'^tionTerm",] PB)ICE TWOPgffiCji,

				

